# 

**Published:** 1893-06

**Authors:** 


					﻿Vol XIII.
JUNE, 1893.
NO. 6X
She
HOMEOPATHIC pHY^lClAfl,
A Monthly Journal of Homoeopathic Materia Medica
and Clinical Medicine.
“ He it a freeman whom truth makes free, and all are slaves beside."
“ Seek the Truth: come whence it may, cost what it unit."
EDITED AND PUBLISH KJ) BY
WALTER M. JAMES, M. D.
‘PHILADELPHIA :
No. 1125 SPRUCE STREET.
LONDON:
ALFRED HEATH & CO., Sole Agents for Great Britain,
114 Ebury Street, Eaton Square.
1 early Svbsfnptiov, $2.60, invariably in advance. Single nnnibtrtf 2 5 ctf,
Entered at the Philadelphia P oat-Office at Second Claan Mail Mauer.
				

